# Supplementary material for: Patterns of Intron Gain and Loss in Fungi
Source: PLoS Biol. 2004 Nov 30;2(12):e422. doi: 10.1371/journal.pbio.0020422 (PMC532390; doi:10.1371/journal.pbio.0020422)
Supplement: Table S1 — Also available at http://genes.mit.edu/NielsenEtAl/. (4.3 MB ZIP). [file pbio.0020422.st001.zip › NielsenEtAl/html/774.html]

AN0558.1.NCU06781.1.MG06722.1.FG06037.1


```
 CLUSTAL W (1.82) Multiple Sequence Alignments - Introns Inserted


Sequence 1: NCU06781.1	494 aa
Sequence 2: MG06722.1	485 aa
Sequence 3: FG06037.1	491 aa
Sequence 4: AN0558.1	550 aa
Alignment Length: 559 aa
Number Identitical Residues: 160 aa
Alignment Score (without introns) 9077


MG06722.1 	-ML0VKSSLLALG--AASIAAAVKPVVVKDNHFVNPENGSRFQIIGVA~YQPGGSAGYKP
NCU06781.1	-ML0IQSTLIAL---SATLAAAVKPLTVKNQFFVDP-NDNIFQIVGVA~YQPGGSAGYNA
FG06037.1 	-ML0INAALMAL---GAAVVGAVPPLEIQGTDFVNPETGNKFQIVGMA~YQPGGSAGYDP
AN0558.1  	MIP0TYGRILAAACALATTASAVTPIEVKGKDFVNSKTGDRFQILGVD2YQPGGTNAVDG
          	 :    . ::* ..: *: ..** *: ::.  **:..... ***:*:  *****: . . 

MG06722.1 	ESGIDPLSNADVCLR0DAALIQSLGANAIRVYNLNPNLNHDECASIFNA0AGIYMVLDVN
NCU06781.1	ALGKDPLSDGDICRR~DAAILQILGVNTIRVYNLNPDVNHDECASIFNA~AGIYMILDVN
FG06037.1 	ATGKDPLSNKDKCMR~DAALMQILGINAIRVYNLDPNVNHDECASIFNA0AGMYMMIDVN
AN0558.1  	KS--DPLSDREACLR~DAALMQQLGVNTIRIYNLSPKLNHDECVSIFNA~AGIYMILDVN
          	    ****: : * * ***::* ** *:**:***.*.:*****.***** **:**::***

MG06722.1 	SPLVGESITSHNPWESYYGSYVNRTLAVVEAFKDYPNTLAFFSGNEVIANVDTGATVPPY
NCU06781.1	SPQVAGSLQNYNPWESYYDTYVNRTFAVVEAFKNYDNTLAFFSGNEVINDASSAGQVPPY
FG06037.1 	SPLPHESLHSLAPWESYHADYLNRTFAIVEAFGNYPNTLLFFAANEVINDEKTAEDAPQY
AN0558.1  	SPLYPGYIDRTAPWTTYTKDYYTQVFGIIEAFKDYPNTLAFFAGNEVINE-DSVEQVPQY
          	**     :    ** :*   * .:.:.::*** :* *** **:.**** : .:   .* *

MG06722.1 	IR~AVTRDLKNYIAKHSKRPIPVGYSAADVRSVLFDSFEYFQCAIDGKSDDMSRADLFAL
NCU06781.1	MR~AVTRDIKNYVAKHCDRKIPVGYSAADVRDVLFDSFEYFTCAEDGKSDDPSRADIFAL
FG06037.1 	IR~ALTRDLKNYVKNNLKRKIPIGYSAADVREVLWDTWNYLQCSDPEDEGDMSRADLFAL
AN0558.1  	IR0AVQRDMKEYISKHLDREIPVGYSAADVRPILEDTLNYFMCEDEDYPN--SSSDFFGL
          	:* *: **:*:*: :: .* **:******** :* *: :*: *      .  * :*:*.*

MG06722.1 	NSYSWCG-DSSFTKSGYDQLVAGFKGTSVPVFFSEYGCNTPSPRIFTEVGAIYGDQMNTV
NCU06781.1	NSYSWCG-DSDMQKSGYVDLVEGFSNTSVPVFYSEYGCNEVKPRMFTEVGAIYGKDFSQV
FG06037.1 	NSYSWCGPEATYETSSYNDLVAGFESSSVPIFFSEYGCIEPAPRYWNETQAIYGKQMSSV
AN0558.1  	NSYSWCG-DASYKSAGYDVLTEMMSEATIPVFFSEYGCNEVQPRKFTEVQAIYGEEMTQT
          	******* ::   .:.*  *.  :. :::*:*:*****    ** :.*. ****.::. .

MG06722.1 	FSGGVVYEYVQEPNNFGLVEINN-DGSVTVLDDYFTLKDQIAKLDFKKVQGVS-AAAGQS
NCU06781.1	FSGGIVYEYTEEENSYGLVSVNTKDQSVTLLKDFYTLKDQFAKLDWKKIQGVKSAKDGSG
FG06037.1 	FSGGVVYEYTEEENNYGLVKIDD--DKVRILGDFNRLKNQFARIKWESVQSNP-AGKNSS
AN0558.1  	FSGGLVYEYTQEENDYGLVKINN-NDTVTLLVDFDNLQTQYAKLDMDRISASN--ASQTS
          	****:****.:* *.:***.::  . .* :* *:  *: * *::. . :..        .

MG06722.1 	AAPPKCDTGLIKEKGFNNNFTLPVPPPGVPEMLQDGIKPAPVGKLVDIKSWKVTHPIKTK
NCU06781.1	PKPPKCDSKLIQSKDFDGNFTLPAMPPNVPKILANGVSPKPSGKLIEVTNFKSKYTVKNA
FG06037.1 	AKAPECKSSLIKDSNFDSNFTIPDVPPKAQGLIDNGIKNKPSGKIVKISDWNVKLDVMNA
AN0558.1  	VKPIKCSADLIKNGTFSSNFTLPAKPPGVQDLINNGYSKVDAGKLVDVESEEVSTTIYDH
          	  . :*.: **:.  *..***:*  ** .  :: :* .    **::.: . : .  :   

MG06722.1 	AGKSIDGLEVKKLANDAINQ~PGSNSG----TGSGTPSSGASAGG---------------
NCU06781.1	DGSVISDLAVKPLSNDESNS1PGTNDAKIQSSGSVTSSSGNSTTS---------------
FG06037.1 	DGSAMKNLKVVPLKDDESNA~SGNNDAD---TGSETTDDKNSTDSD--------------
AN0558.1  	NGKEITGLKLTVLADGESNQ~PGSSSIGSSSNSGSDSDSGDDDNAAGIVAIPVGLATIAA
          	 *. : .* :  * :.  *  .*...   .:...  ...  .  . .  : . . :: ::

MG06722.1 	----~-------------------~ASASPDK~KDAAGNVRVGFATVFVSAAAAAACAVF
NCU06781.1	----~-------------------~GSSSEAT~SDAKNTSGAAGSAVAMTVPAMAMGAFA
FG06037.1 	----~----------------NKT~SSSSGDA~KDGDDDEDAAGLNRPLAWAMAFPLAVM
AN0558.1  	TLFD1NVSLGLYYTSQMFSSLQDY2TSVVGRL1TSSKSDRTTNGAGYLPEPSVFFSVRYH
          	:  . . : .   ::.  :: ..   *      ... .   .         .        

MG06722.1 	VF------~--------------------
NCU06781.1	IFAGLAL-~--------------------
FG06037.1 	MFAL----~--------------------
AN0558.1  	MFEEVLII2LNMFMAALEALKFDLTLQLK
          	:*        .   :: .: . . : . .
```
